# Engineering viral genomics and nano-liposomes in microfluidic platforms for patient-specific analysis of SARS-CoV-2 variants

**DOI:** 10.7150/thno.72339

**Published:** 2022-06-06

**Authors:** Sandro Satta, Fahimeh Shahabipour, Wei Gao, Steven R. Lentz, Stanley Perlman, Nureddin Ashammakhi, Tzung Hsiai

**Affiliations:** 1 Department of Bioengineering, School of Engineering, University of California, Los Angeles, California, USA.; 2 Skin Research Center, Shahid Beheshti University of Medical Science, Tehran, Iran.; 3 Medical Engineering, California Institute of Technology, California, Pasadena, USA.; 4 Department of Bioengineering, Henry Samueli School of Engineering & Applied Science, University of California, CA, USA.; 5 Institute for Quantitative Health Science & Engineering and Department of Biomedical Engineering, College of Engineering, Michigan State University, MI, USA.; 6 Department of Medicine, Greater Los Angeles VA Healthcare System, Los Angeles, California, USA.; 7 Division of Cardiology, Department of Medicine, School of Medicine, University of California, Los Angeles, California, USA.; 8 Section of Hematology, Oncology, and Blood & Marrow Transplantation, Department of Medicine, College of Medicine, University of Iowa, Iowa, USA.; 9 Department of Microbiology and Immunology, College of Medicine, University of Iowa, USA.

**Keywords:** Organ on-a-chip, microfluidics, viral genomics, mutations, variants of concerns, COVID-19, nano-liposomes

## Abstract

New variants of severe acute respiratory syndrome coronavirus 2 (SARS-CoV-2) are continuing to spread globally, contributing to the persistence of the COVID-19 pandemic. Increasing resources have been focused on developing vaccines and therapeutics that target the Spike glycoprotein of SARS-CoV-2. Recent advances in microfluidics have the potential to recapitulate viral infection in the organ-specific platforms, known as organ-on-a-chip (OoC), in which binding of SARS-CoV-2 Spike protein to the angiotensin-converting enzyme 2 (ACE2) of the host cells occurs. As the COVID-19 pandemic lingers, there remains an unmet need to screen emerging mutations, to predict viral transmissibility and pathogenicity, and to assess the strength of neutralizing antibodies following vaccination or reinfection. Conventional detection of SARS-CoV-2 variants relies on two-dimensional (2-D) cell culture methods, whereas simulating the micro-environment requires three-dimensional (3-D) systems. To this end, analyzing SARS-CoV-2-mediated pathogenicity via microfluidic platforms minimizes the experimental cost, duration, and optimization needed for animal studies, and obviates the ethical concerns associated with the use of primates. In this context, this review highlights the state-of-the-art strategy to engineer the nano-liposomes that can be conjugated with SARS-CoV-2 Spike mutations or genomic sequences in the microfluidic platforms; thereby, allowing for screening the rising SARS-CoV-2 variants and predicting COVID-19-associated coagulation. Furthermore, introducing viral genomics to the patient-specific blood accelerates the discovery of therapeutic targets in the face of evolving viral variants, including B1.1.7 (Alpha), B.1.351 (Beta), B.1.617.2 (Delta), c.37 (Lambda), and B.1.1.529 (Omicron). Thus, engineering nano-liposomes to encapsulate SARS-CoV-2 viral genomic sequences enables rapid detection of SARS-CoV-2 variants in the long COVID-19 era.

## Introduction

Coronavirus disease 2019 (COVID-19) has inflicted more than 400 million people, resulting in more than five million deaths, and new variants are relentless 1-3. Despite the newly released vaccines to target the Spike glycoprotein, SARS-CoV-2 variants have evolved with several mutations that evade the neutralizing antibodies in the vaccinated populations 2, 4, 5. The recent rise in Spike mutations, including B1.1.7 (alpha) and the rapid spread of viral variants, including B.1.351 (Beta), c.37 (Lambda), B.1.617.2 (Delta), and B.1.1.529 (Omicron), have met with persistent public health crises and the urgent need for coverage by vaccines and therapeutic agents 1. Conventional strategies to screen and intervene with SARS-CoV-2 infection have been met with rapid mutations and increasing transmissibility and pathogenicity of new variants 6, 7. The current drug development relies on the two-dimensional (2-D) cell culture systems that negate the pathophysiological events occurring in a three-dimensional (3-D) environment 8, and the primate models require experimental optimization, increased resources and ethical concerns 9. Furthermore, numerous viral pathogens are species-specific, and the results obtained from animal experiments may not be clinically translational to humans 10. In this context, this review focuses on engineering the SARS-CoV-2 genomics in the organ-specific microfluidic platform, also known as organ-on-a-chip (OoC), thus providing an alternative but efficient strategy to detect SARS-CoV-2 variants and to assess therapeutic targets 4. To this end, introducing viral genomics to the patient-specific blood in the microfluidic platforms holds promise to recapitulate the interplay between patient-specific tissue/organ and pseudo-SARS-CoV-2 viral particles 11, 12. We will demonstrate the OoC technology to investigate the tissue-specific viral infection, introduce multi-channel arrays for screening COVID-19 mutations and for predicting COVID-19-associated coagulation. This review will further highlight how engineering SARS-CoV-2 genomic sequences, Spike protein variants, and human angiotensin-converting enzyme 2 (hACE2) decoys can be applied to study COVID-19-associated coagulation. The goal is to rapidly detect and countermeasure the rising SARS-CoV-2 variants for personalized medicine.

## Microfluidic platforms for viral infection

**Figure 1:**
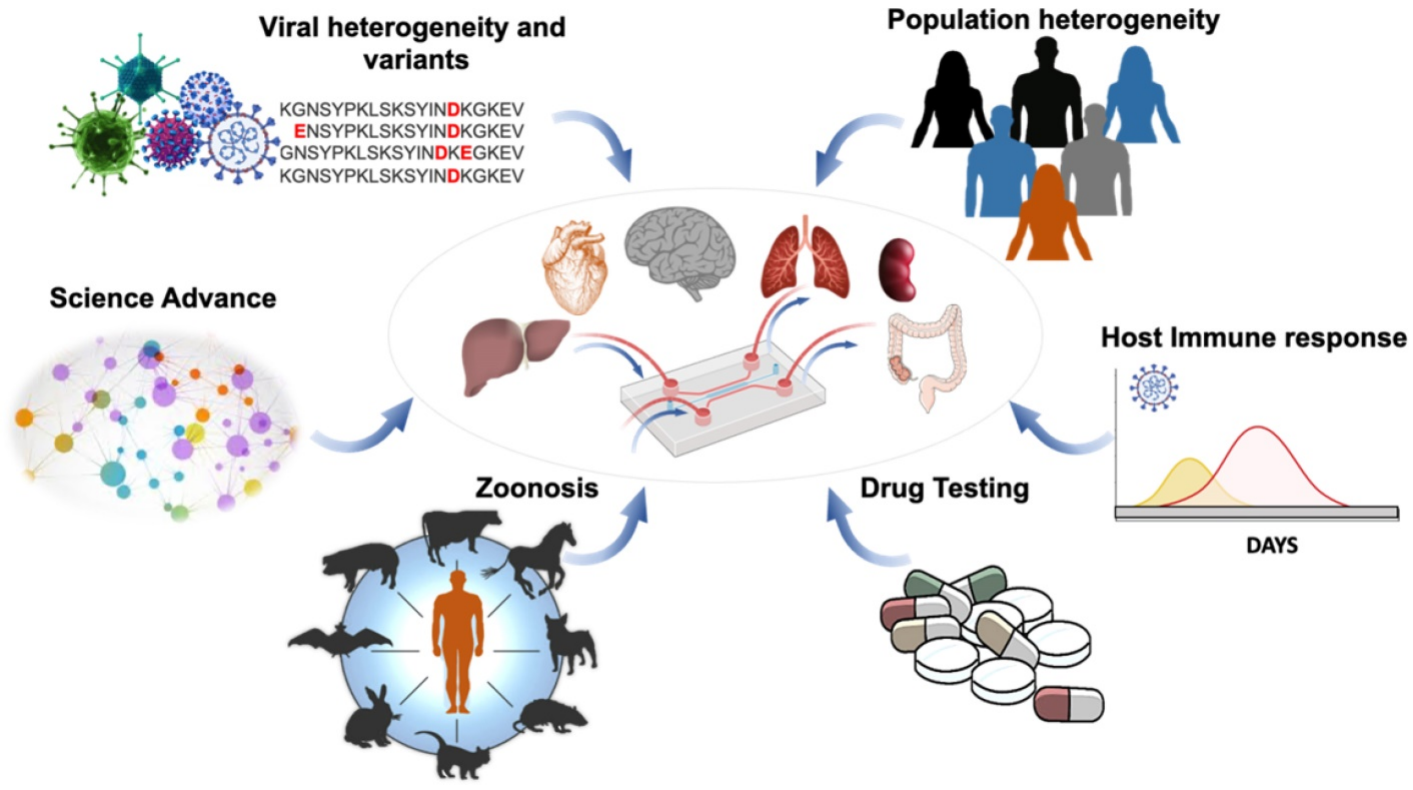
**Organ-on-a-chip for viral infection**. Application of OoC affords an opportunity to uncover virus mutation and transmission, as well as patient-specific analyses and therapeutic targets. This illustration highlights the capacity of the integrated microfluidic platform to simulate organ-specific tissues for (1) screening the SARS-CoV-2 heterogeneity and variants, (2) identifying the population heterogeneity with different susceptibilities to SARS-CoV-2 variants, (3) detecting animal-to-human transmission (zoonosis), (4) analyzing the host immune responses, including the antibody titers against SARS-CoV-2 variants, and (5) performing bioinformatics for drug development and repurposing Adapted from 23, with permission from Trends Microbiol, copyright 2020. Created using BioRender.com.

Amidst the resurgence of the SARS-CoV-2 variants across the globe, the scientific community has increased surveillance to identify mutations in the circulating SARS-CoV-2 variants that might increase infectivity, enhance pathogenicity, or alter coverage by drugs or vaccines 1. OoC allows for simulating the tissue-specific microenvironment (**Figure 1**) 13, 14 in which one functional unit of the target organ or tissue enables patient-specific molecular or cellular analyses 14-16. The OoC systems afford microvascular circulation in which cells are exposed to fluid shear stress and patient's blood components, including coagulation factors, cytokines, immune cells, and platelets 17. Micro-devices, including mechanical valves, electrodes, and sensors, can be embedded in the OoC systems for studying response to SARS-CoV-2 infection. The OoC unit can be tailored to the organ-specific anatomy and function 18. OoC is microfabricated from an optically transparent biomaterial well-known as polydimethylsiloxane (PDMS) which allows gaseous diffusion, including oxygen (O_2_) and carbon dioxide (CO_2_) 19, 20. PDMS is conducive to seeding the primary human cell lines interacting with the freshly isolated human blood mononuclear cells (HBMC) or stem cells in response to the SARS-CoV-2 pseudovirus 21, 22. Thus, the microfluidic platforms provide high-throughput analyses with minimal animal cost and experimental duration; thus, obviating the ethical concerns for the use of animal models.

## Microfluidic platform for SARS-CoV-2 Spike protein

**Figure 2:**
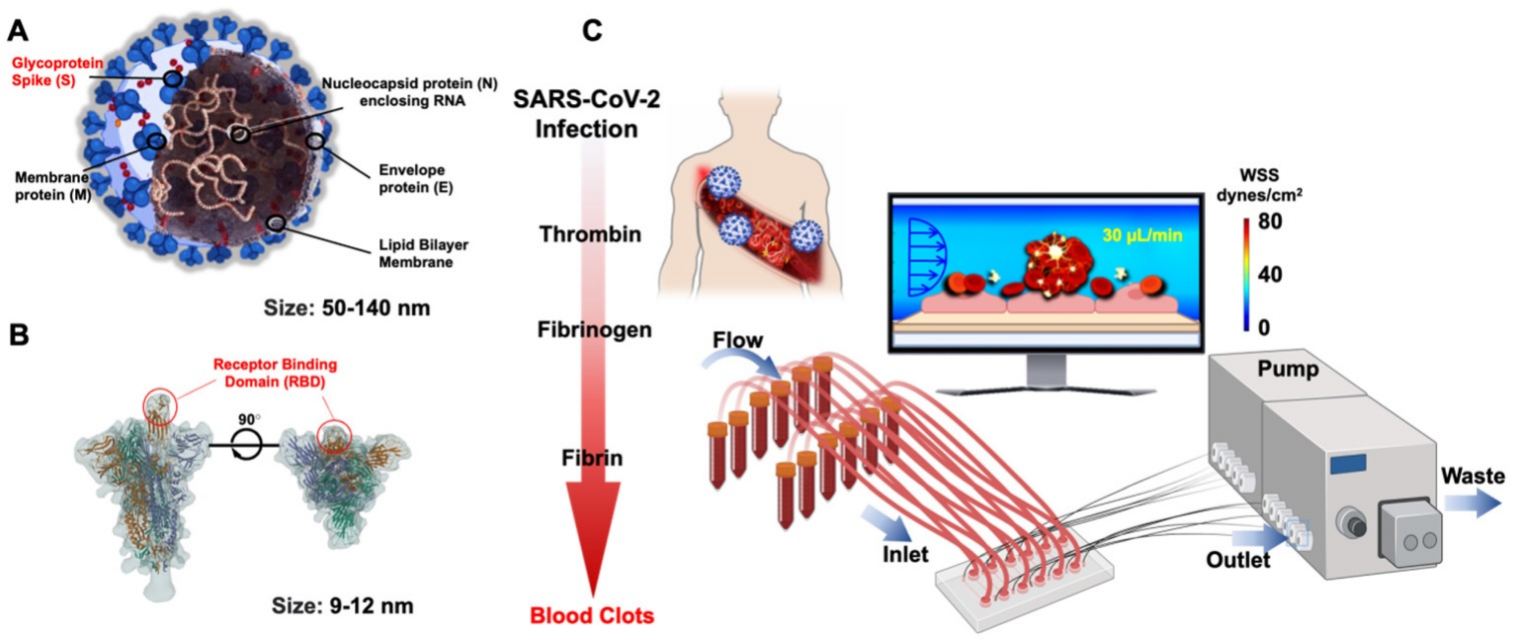
**Structural proteins of SARS-CoV-2 in microfluidic platforms**. (**A**) Schematic structure of the Spike (S) protein in relation to envelope (E), membrane (M), and nucleocapsid (N) proteins. (**B**) Spike protein is composed of S_1_ and S_2_ subunits. The RBD of S_1_ protein binds to the hACE2 receptor. (**C**) In response to viral infection, inflammatory cells are recruited to activate the coagulation pathways, leading to blood clot formation or known as thrombosis. The multi-array microfluidic platform enables high-throughput screening of patient-specific COVID-19-associated coagulation in response to pseudo-SARS-CoV-2 inoculation, obviating the need to perform the studies in the viral containment Biosafety Laboratory 3 (BSL3). Created using BioRender.com.

SARS-CoV-2 belongs to a group of a previously known family of coronaviruses, and its structural proteins provide the molecular basics to develop therapeutic targets and vaccinations. Of the structural proteins, the membrane protein (M) is the most abundant structural protein for the spherical shell. The envelope (E) and nucleocapsid protein (N) enclose the viral genome. The Spike protein (S) extends from the virus and comprises the crown of thorns that bind to the hACE2 expressed by the host cells 24 (**Figure 2A**). In the face of the lingering COVID-19 pandemic, intense research has targeted on the binding affinity between the Spike protein and human ACE2 25, 26. The cellular entry of SARS-CoV-2 is achieved by the homotrimeric Spike-mediated virus-receptor engagement through the receptor-binding domain (RBD) binding to hACE2, followed by virus-host membrane fusion 17, 27-29 (**Figure 2B**). The rising mutations in the Spike protein alter the SARS-CoV-2 transmissibility, fusogenicity, and pathogenicity 21. Once internalized into the host cells, the cytoplasmic membranous structures of the virus are assembled into replication vesicles 22, 30. For this reason, the common therapeutic target is to inhibit viral internalization and transcription into the host cells by activating the host immune responses against the Spike protein 31, 32. However, the rise in Spike mutations, including B1.1.7 (Alpha) and the rapid spread of viral variants, including B.1.351 (Beta), B.1.617.2 (Delta), c.37 (Lambda), and lately Omicron 33, is a testimony to an increased viral transmissibility, that evades the neutralizing antibodies in vaccinated individuals 34, 35. In this context, inhibition of the binding affinity of the Spike protein to the ACE2 has been the focus for therapeutic targets, and rapid-COVID-19-on-a-chip has provided high-throughput analyses using the patient-specific whole blood and pseudo-SARS-CoV-2 virus. Integrating the SARS-CoV-2 genomics with the microfluidic platforms allow for a multi-array detection of COVID-19-associated coagulation, avoiding direct viral exposure (**Figure 2C**). Furthermore, COVID-19-on-a-chip affords a non-animal model to screen therapeutic targets in the face of evolving viral variants.

## Engineering virus-on-a-chip for organ-specific systems

Microfluidic platforms are ideal to elucidate the infectivity of the virus (~100 nm in diameter) and to determine the efficacy of therapeutic targets 36-38. Guo *et al.* developed a microfluidic platform mounted on a fluorescence microscope, allowing for monitoring the kinetics of viral infection throughout the entire life cycle in terms of viral replication rate and yield 39. Thus, the use of OoC facilitates the studies of virus-host interactions.

### SARS-CoV-2 in the lung-on-a-chip

The lung is the primary target of SARS-CoV-2 infection 40, and the alveolus is the main functional unit in the lungs. The alveolar-capillary barrier maintains gas exchange and prevents viral entry. Diffuse alveolar damage and overwhelming inflammation develop in the setting of severe COVID-19 infection, leading to acute respiratory distress syndrome (ARDS) 41, 42. Thus, lung-on-a-chip models allow for recapitulating immune responses during the cytokine storm in the lung 43.

**Figure 3:**
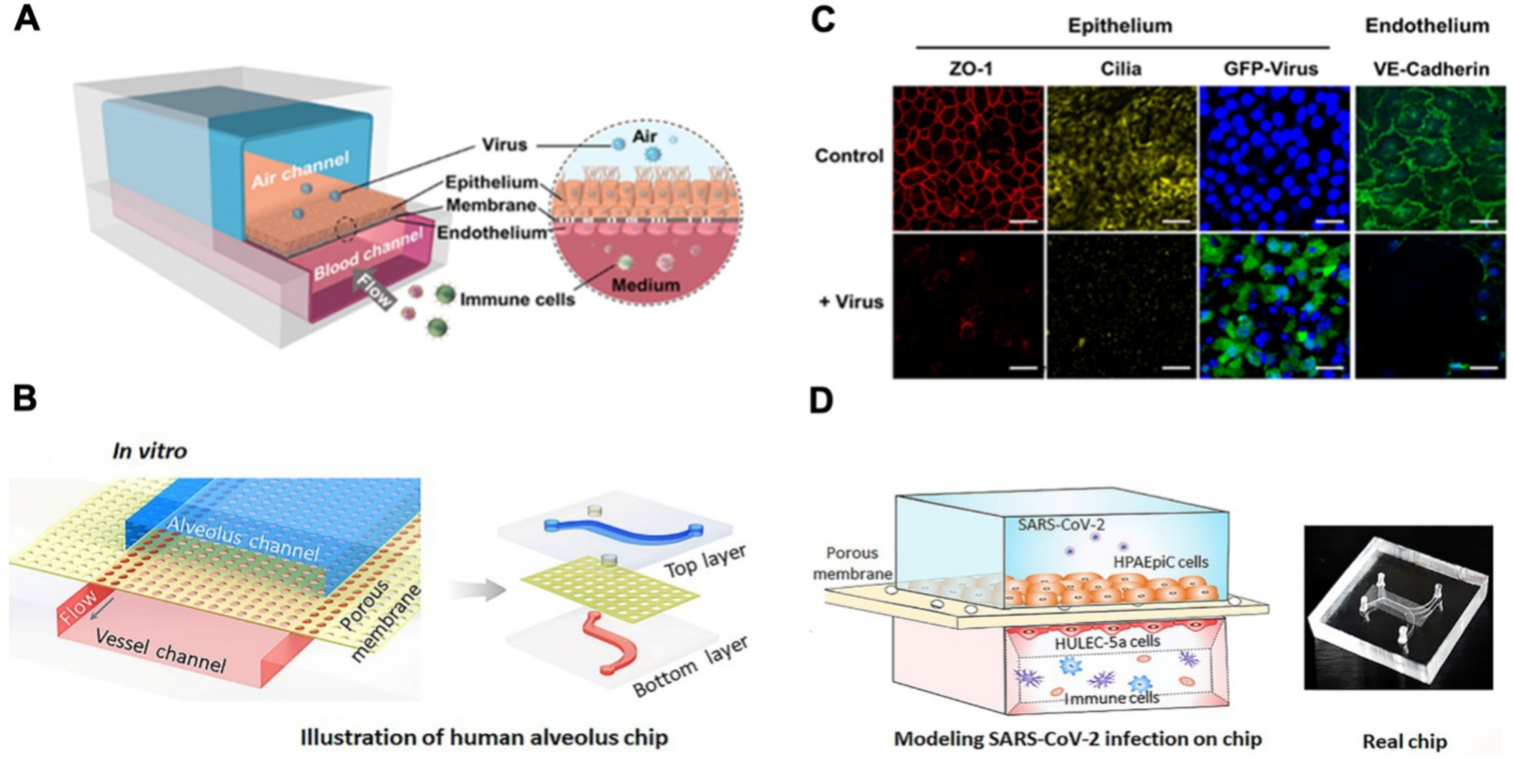
**Lung-on-a-chip.** (**A**) Schematic diagram of human airway chips. (**B**) Immunofluorescence micrographs of cells: ZO-1 shows tight junctions, cilia in the epithelium, and VE-cadherin in the junctions of the endothelium in the airway-on-a-chip without (Control) and without virus (+ Virus) for 48 h (Blue, DAPI: stained nuclei, Scale bar is 50 µm). Reproduced from 47, with permission from Cold Spring Harbor Laboratory. (**C**) Schematic diagram represents configuration of human alveolus-on-a-chip infected by SARS-CoV-2. The chip is divided into two chambers by a PDMS membrane: upper alveolar epithelial chamber (blue) and lower pulmonary microvascular endothelial chamber (red). (**D**) The alveolar-capillary boundary is formed by co-culture with alveolar epithelial cells (HPAEpiC) and pulmonary microvascular endothelial cells (HULEC-5a) under flow condition. The constructed alveolus chip is exposed to SARS-CoV-2 via the epithelial layer. After virus infection, human immune cells are infused into the vascular chamber. Image of the chip is shown. Reproduced from 43, with permission from Wiley, copyright 2020.

A human lung airway-on-a-chip can be micro-fabricated with the extracellular matrix (ECM) coated with the porous membrane to separate airways from the vascular channels (**Figure 3A & B**) 44. Lung epithelial cells in the airway-on-a-chip express ACE2 to promote binding and the transmembrane protease serine-2 (TMPRSS2) to facilitate fusion of SARS-CoV-2 into the host cells. Human lung airway-on-chip can further be used to screen the Federal Drug Administration (FDA)-approved drugs for treating SARS-CoV-2 infection, and the microfluidic system compartmentalizes the alveolar lumen from the microvascular chamber via a thin PDMS membrane (~25 μm) (**Figure 3C & D**) 43, 45, 46. The alveolar chamber is seeded with human alveolar epithelial type II cell (AT II) lines (HPAEpiC), and the vascular chamber with lung microvasculature cells (HULEC-5a). When SARS-CoV-2 is inoculated into the alveolar chamber, the Spike proteins are recognized by the epithelial cells. Immune responses can be assessed by infusing human peripheral blood mononuclear cells (PBMCs) into the vascular chamber, where cytokine expressions, including IL-1β, IL-6, IL-8 and TNF-α, can be detected. This human alveolus-on-a-chip can also demonstrate the feasibility to repurpose FDA-approved remdesivir as a therapeutic agent to restore epithelial and endothelial dysfunction 43.

### SARS-CoV-2 in the gut on-a-chip

**Figure 4:**
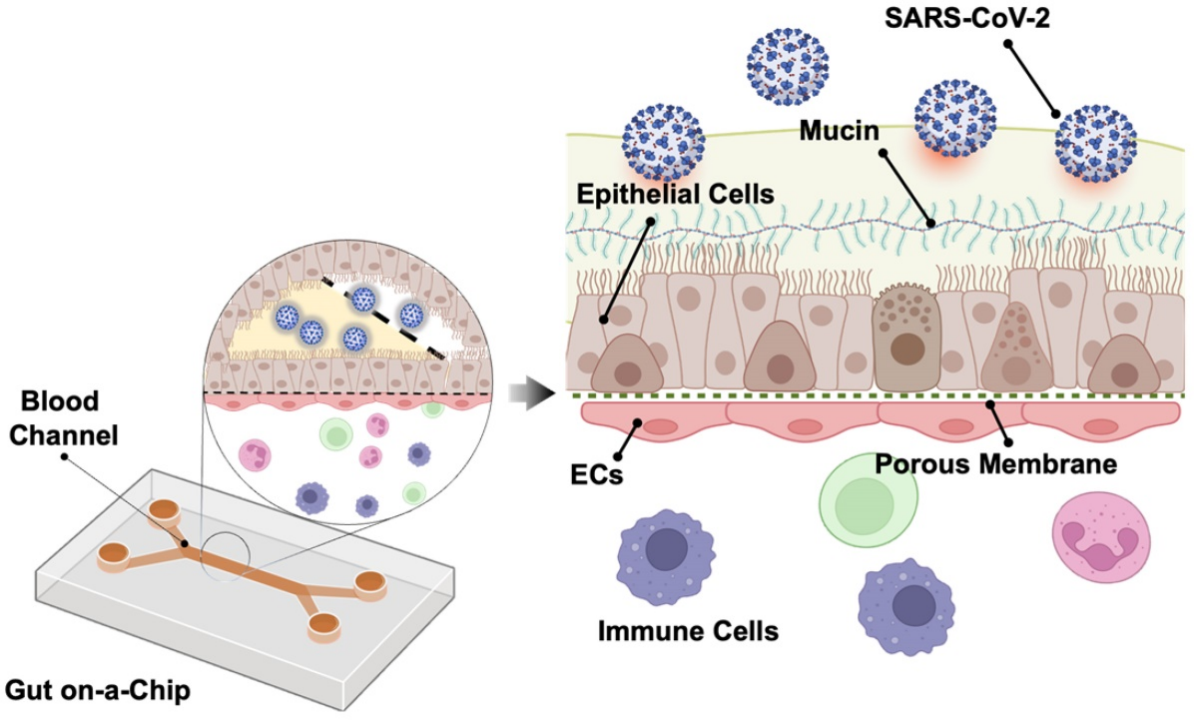
** Gut-on-a-chip.** The chip has two chambers separated by an ECM coated with the PDMS membrane. The upper chamber is seeded with the intestinal epithelial cells (Caco-2 and mucin-secreting HT-29 cells) and the lower chamber with human umbilical vein endothelial cells (HUVECs). SARS-CoV-2 is inoculated into the upper chamber. Reproduced from 50, with permission from Am J Gastroenterol, copyright 2020. Created using BioRender.com.

Human intestine is recognized as one of the entry points for SARS-CoV-2 infection 48. Intestine comprises an important part of the human immune system, with a large population of innate and adaptive effector cells. Due to its constant exposure to the foreign and environmental antigens, intestine is covered by a layer of protective mucus gel secreted by the epithelial cells as the chemical and physical barrier in the intestine 49. Following SARS-CoV-2 infection, Guo *et al.* observed that mucin secretion started from an agglomerated to a scattered distribution in the biomimetic human gut-on-chip system 50. Human gut on-a-chip has an intact intestinal barrier that is composed of villus-like structures along the apical surfaces, together with a mucus-secretion function under fluidic flow (**Figure 4**). Herein, intestinal epithelium-endothelium interactions resemble the gut-vascular barrier in human intestine. After SARS-CoV-2 infection, disruption of the villi is observed, along with the intestinal barrier integrity and mucus secretion. In the setting of activated immune cells, the vascular endothelium can also develop morphological damage. These observations may explain the mechanisms underlying the increased intestinal permeability and COVID-19-associated diarrhea and hemorrhagic colitis 51.

## Integrating SARS-CoV-2 genomics in the microfluidic platforms

### Integrating SARS-CoV-2 with vascular endothelium-on-a-chip to predict COVID-19-associated coagulation

Due to genetic heterogeneity in the population, there remains a paucity of patient-specific screening for SARS-CoV-2-associated coagulation, also known as thrombosis 52, 53, that manifests as deep vein thrombosis, pulmonary embolism, and stroke. Microfluidic systems with small perfusion volumes can replicate platelet aggregation and fibrin degradation in the vasculature, in which fluid shear stress or blood flow promotes blood coagulation 54. Unlike the existing methods relying on the murine and primate models 55, 56, vascular endothelium-on-a-chip allows for screening SARS-CoV-2 Spike variants or COVID-19 blood in the setting of endothelial inflammation, complement activation, thrombin generation, platelet and leukocyte recruitment, along with immune responses 57-59. Thus, microfluidic platforms create a microenvironment in which vascular endothelial cells are exposed to the mutation of SARS-CoV-2 Spike proteins 60. Increasing evidence supports an “immunothrombosis” sequalae following recent viral infection. Notably, these thrombotic events delay fibrinolysis, increase von Willebrand factor (vWF) and factor VIII levels, and generate the “lupus antiphospholipid antibodies” 61-68. As a majority of COVID-19 patients are prophylactically anticoagulated during the acute phase of hospital admissions, there remains an unmet clinical need to predict whether the long COVID-19 patients are predisposed to developing thrombotic events 69. Therefore, integrating pseudo-COVID-19 or SARS-CoV-2 Spike variants and vascular endothelium-on-a-chip offers the opportunity for timely thromboprophylaxis during both the acute and post-acute sequalae of COVID-19 70.

**Figure 5:**
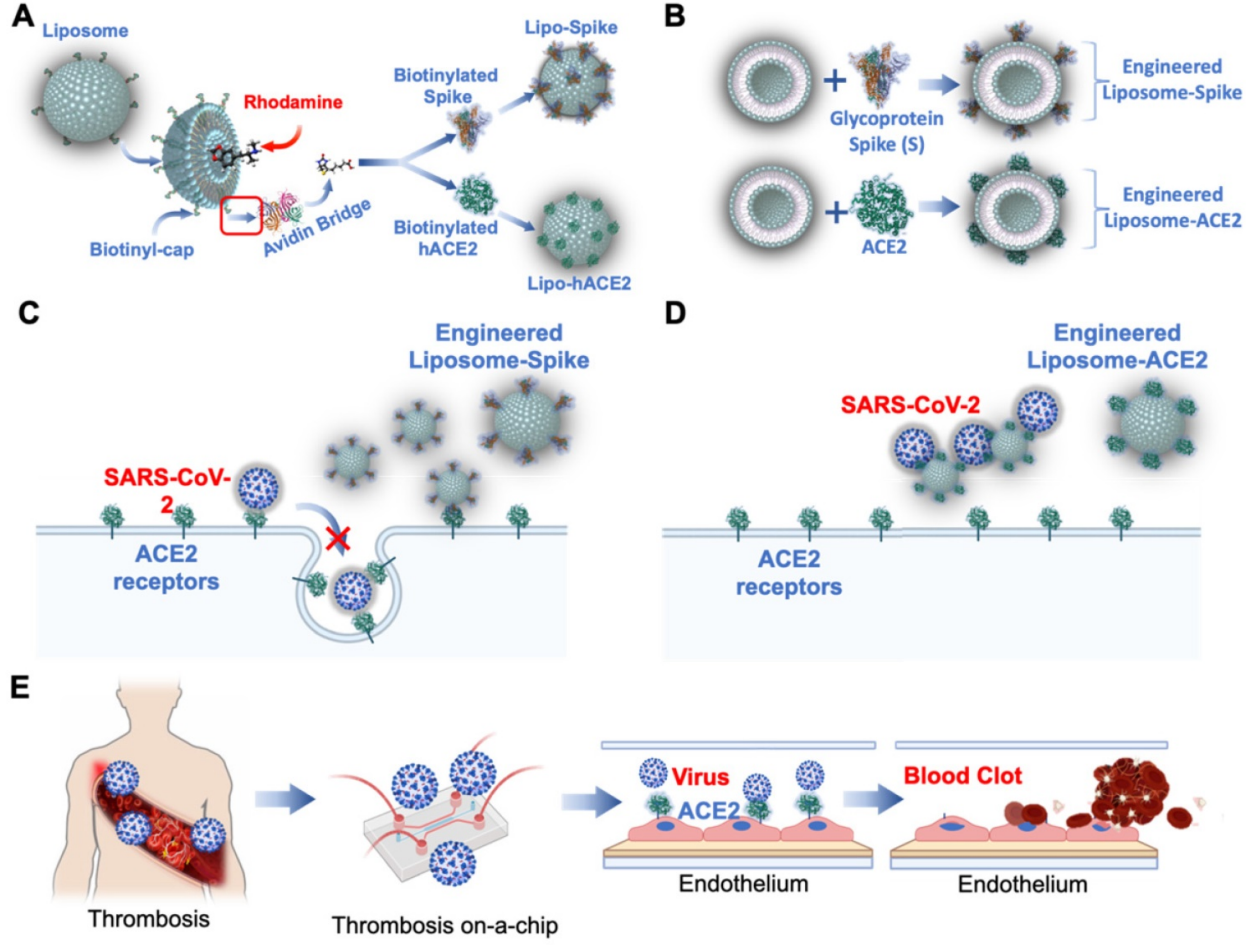
**Engineering Lipo-Spike and Lipo-hACE2**. (**A**) Fluorochromes such as coumarin-6 or rhodamine are encapsulated in the liposomes during self-assembly. Biotinylated liposomes are conjugated non-covalently (d = 100 nm) with Neutravidin protein to form Liposomes-biotinyl Cap-Neutravidin (88). (**B**) Next, biotinylated SARS-CoV-2 S-protein, His, Avitag (Acrobiosystem), are bound to the Liposomes-biotinyl Cap-Neutravidin, forming Liposome-Spike conjugation. hACE2 (Acrobiosystem: Biotinylated Human ACE2/ACEH Protein, Fc, Avitag AC2-H82F9-25ug) is biotinylated with Liposomes-biotinyl Cap-Neutravidin, forming liposome-hACE2 conjugation. **Lipo-Spike competes with SARS-CoV-2 for the host ACE2 receptors**. (**C**) Recombinant-Glycoprotein-S-Spikes are biotinylated to the liposomes, promoting binding to the ACE2 receptors expressed on the host cell membrane; thus, competing with SARS-CoV-2 for internalization into the host cells. (**D**) **Lipo-ACE2 decoy competes with SARS-CoV-2 for the host ACE2 receptors**. Recombinant-ACE2 proteins are biotinylated to the liposomes, promoting SARS-CoV-2 binding to the liposomes; thereby, preventing SARS-CoV-2 from binding to the host ACE2 receptors. (**E**) **Patient-specific blood is used to predict COVID-19-Associated Coagulation**. A schematic of the human artery endothelial cells (HAEC)-seeded microfluidic chip mimics liposome Spike entry into the endothelial cells. Reproduced from 89, 90, with permission from Wiley and Theranostics, copyright 2021-2022. Created using BioRender.com.

As a corollary, thrombotic events are reported to have occurred following adenovirus and mRNA-based vaccines 71-79. Vascular endothelium-on-a-chip is conducive to predicting COVID-19-associated coagulation in both vaccinated and previously infected individuals. Patient-specific blood can be screened in the presence of nano-liposomes (diameter = 10 nm) that are biotinylated with functional S-Spike (Lipo-S) 80, and blood clot inhibition can develop with the nano-liposomes conjugated with human ACE2 (Lipo-hACE2) as a decoy for the Spike proteins (**Figure 5A-B**) 81, 82. As viral infection activates the immune system, endothelial injury and complement-induced coagulopathy 83-85, microfluidic systems can be used to simulate these events for personalized identification of Spike variant-mediated thrombosis (**Figure 5C-E**) 86, 87.

### Engineering SARS-CoV-2 sub-genomic sequences to screen variants and thrombosis

**Figure 6:**
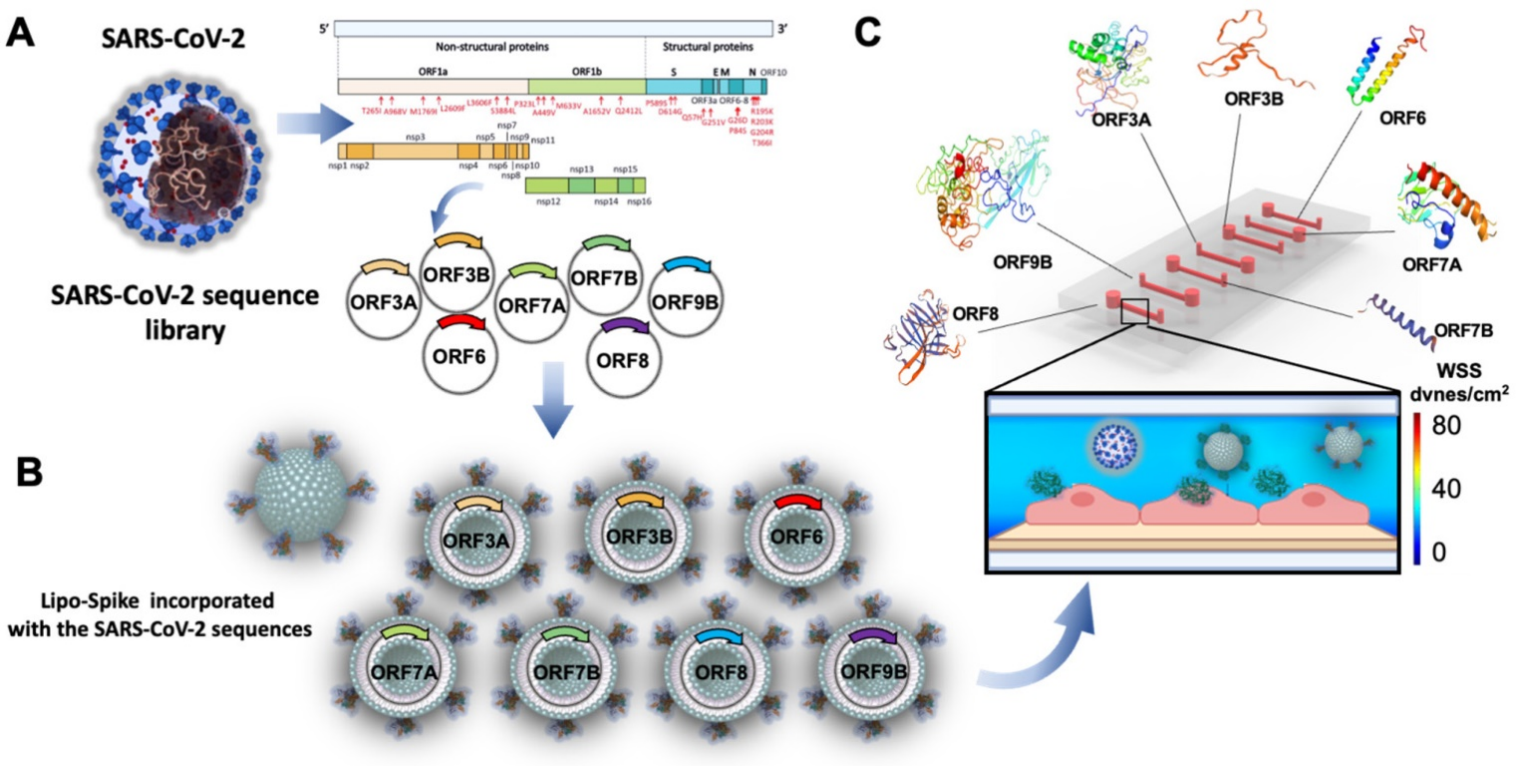
**Engineering SARS-CoV-2 genomes to screen COVID-19-associated coagulation**. (**A**) Sub-genomic and SARS-CoV-2 Spike sequences are cloned and inserted into the nano-liposomes. Specific regions of NSP 1-16) for 5' untranslated region (UTR) and open reading frame (ORF 3, 6, 7, 8, & 9) for 3' UTR or the CoV-2-Spike can be incorporated into the nano-liposomes that are conjugated with Spike proteins. (**B**) A schematic of SARS-CoV-2 viral nanoparticles highlight the incorporation of genomic sequence-specific into the nano-liposomes. (**C**) Nanoparticles can be infused into a multi-array microfluidic platform for screening a host of genomic sequences, including Spike mutations and variants. Shear stress activated ACE2 receptors facilitate the binding of the nanoparticles to the endothelial cells. Microfluidic channel (400 µm x 100 µm x 2 cm) is seeded with tissue-specific cell types to simulate viral interaction in an organ-specific chip. Created using BioRender.com.

SARS-CoV-2 harbors a large single-stranded RNA genome (30kb) 91. Molecular mechanisms whereby the genome is folded in the virion, together with its regulatory non-structural protein (NSP) and structural proteins (**Figure 6A**), remain unknown 92. Single SARS-CoV-2 sequences have been shown to develop into inflammasome activation 93, interfering with Interferon (IFN) pathways 94 and apoptosis 95. Furthermore, evidence has supported the involvement of autoimmunity in COVID-19 patients 96. Understanding autoimmune and inflammatory responses to these proteins would further uncover how SARS-CoV-2 uses the host-cell machinery for its replication and host signaling pathways for its pathogenicity. To demonstrate personalized strategy to predict COVID-19-associated coagulation (CAC), investigators have demonstrated the insertion of the single SARS-CoV-2 sub-genomic sequences into the nano-liposomes that are conjugated with SARS-CoV-2 Spike proteins during the lipid self-assembly process (**Figures 6A & B**). This integration of nano-liposomes with microfluidics enables rapid screening of SARS-CoV-2 sub-genomic sequences for CAC, lupus antiphospholipid antibodies 61, 63, 68, and inflammatory cytokines. As a corollary, the engineered liposome-Spike proteins 60 are used as a pseudo-SARS-CoV-2 to obviate the need for the real SARS-CoV-2 virus and the requirement for BSL3 (**Figure 6C**).

### Engineering nano-liposomes to access SARS-CoV-2 Spike variants

**Figure 7:**
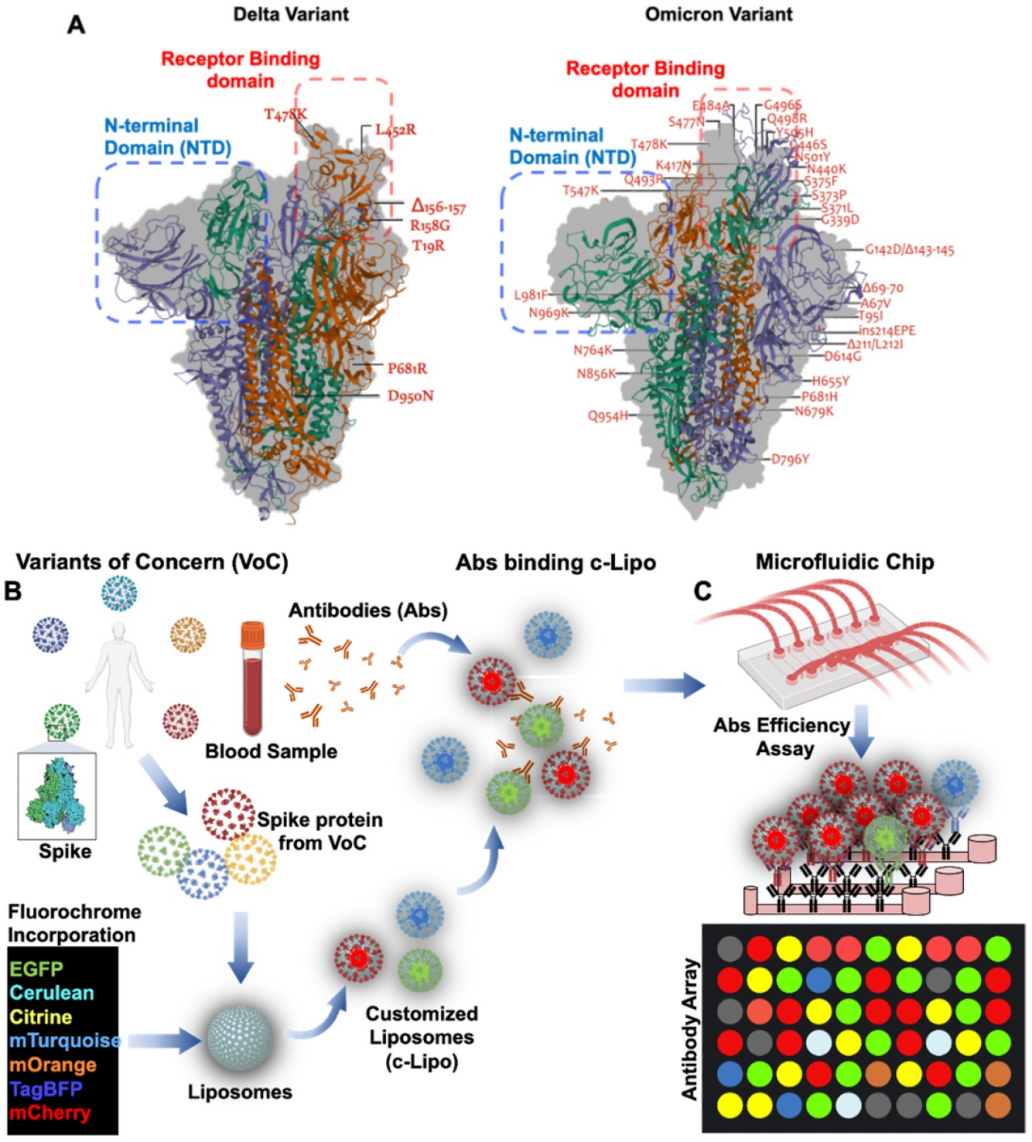
** Mutations arising from SARS-CoV-2 Variants of Concern: Omicron vs. Delta**. (**A**) Q493R, N501Y, S371L, S373P, S375F, Q498R, and T478K mutations contribute to the high binding affinity to hACE2. In comparison to the Delta variant, both the Spike protein and RBD in Omicron are comprised of a high proportion of hydrophobic amino acids such as leucine and phenylalanine. These amino acids are located within the protein's core to provide structural stability of the Spike protein. It is postulated that a disorder-order transition in the Omicron variant between Spike protein RBD regions 468-473 may influence the disordered residues/regions on Spike protein stability and binding to ACE2**. (B) Nab targeting VoC in the microfluidic platforms.** Schematic representation of VoC. Spike proteins derived from the specific VoC can be conjugated with C-Lipo encapsulated with fluorochrome 88. Abs from the vaccinated or recovered COVID-19 blood can be assessed for their efficiency against the specific VoC. (**C**) A mix of c-Lipo and Abs from blood sample can be infused into a multi-array microfluidic platform for ELISA-like detection of VoC. Fluorescent signals can be used to quantify the optimal combination of Ab candidates targeting the VoC. Abs: antibodies; VoC: Variants of Concern. Created using BioRender.com.

Global emergence of SARS-CoV-2 Variants of Concern (VoC) has been the lingering factors of the persistent or recurrent pandemic 97. Variations in amino acids in the Spike protein are considered the mechanisms underlying viral transmissibility and pathogenicity 58. Engineering nano-liposomes conjugated with SARS-CoV-2 Spike mutations would accelerate the investigation of the evading neutralizing antibodies from the SARS-CoV-2 variants, including B1.1.7 (Alpha), B.1.351 (Beta), B.1.617.2 (Delta), c.37 (Lambda), and B.1.1.529 (Omicron) variants (**Figure 7**). SARS-CoV-2 Delta variant was first identified in India 59, and this variant was resistant to neutralization by some anti-N-terminal domain (NTD) and anti-RBD monoclonal antibodies. For example, the FDA-authorized monoclonal antibody for treating COVID-19, bamlanivimab, which was reported to exhibit impaired binding to the Spike protein of Delta 60. This VoC is believed to be 60% more transmissible than the Alpha variant 58. Similarly, the latest VoC Omicron, contains 15 mutations in the RBD, and is capable of evading RBD neutralizing antibodies (Nabs). It has been observed that Nabs, whose epitopes overlap with ACE2-binding motif, might not interact with Omicron RBD due to K417N, G446S, E484A, and Q493R mutations. Additional mutations in the G339D, N440K, and S371L sites contribute to increase Nabs evasion, supporting that Omicron escapes greater than 85% of antibodies tested 98. In this case, the use of microfluidic platforms recapitulates the *in vivo* microenvironment for patient-specific screening for Nabs 44, 56, 99. Unlike the enzyme-linked immunosorbent assay (ELISA), the entire SARS-CoV-2 viral proteome can be translated into overlapping peptides and printed onto the glass slides. The sera from COVID-19 patients can be incubated in the microfluidic platforms and the antibodies present in the patient-specific blood sample can bind to the epitopes expressed by the individual peptides. Testing SARS-CoV-2 Nabs binding affinity further optimizes the spectrum of resistance to immune escape 46. As a corollary, using post-COVID-19 or vaccinated blood allows for recovering Nabs to predict efficiency against the new VoC. By incorporating the fluorochrome in the customized Liposomes (c-Lipo) and conjugating the Spike proteins from VoC, researchers would be able to screen the Nabs targeting the c-Lipo-Spike-VoC. Fluorescence signals from the c-Lipo can be quantified for Nabs efficiency targeting the VoC (**Figure 7**). Thus, integrating the viral genomics with microfluidic platforms allows for screening the patient-specific whole blood; thus, providing an opportunity to detect and prevent against the SARS-CoV-2 variants in a personalized manner.

### Microfluidic chips for repurposing pharmaceutical agents to target SARS-CoV-2

Despite the progress made during the COVID-19 pandemic, the molecular mechanisms underlying SARS-CoV-2-mediated infection are distinct from its predecessor SARS-CoV 47. Two-dimensional (2-D) cell culture models are limited to simulate *in vivo* environment, and experimental animals cannot recapitulate human physiology for drug testing 102, 103. For this reason, an *in vivo* system that can employ patient samples would have the capacity to expedite drug screening for the time-sensitive COVID-19 research 89. To this end, microfluidic platforms enable the clinicians to assess the efficacy of new antiviral drugs alone or in combination with other repurposed drugs 43. The compartmentalization of the microfluidic device allows for precise control of the microenvironment, and separate administration of individual drugs. Also, the microchannels enable sub-compartments to recapitulate multiple cellular or tissue-specific responses. Thus, allowing optimal nutrients and oxygen supply and efficient removal of metabolites at the micro scale 104. For instance, a lung-on-a-chip seeded with the human lung epithelial cells (HLEPCs) allows for expression of both hACE2 and TMPRSS2; thereby, enabling clinicians to assess the inhibitory effects of the repurposed drugs 47. Similarly, a two-channel microfluidic platform was used for seeding intestinal epithelium cells developing into the villus-like structures over 2-3 weeks. Once the villi were established, the apical medium promoted the differentiation of microvascular endothelial cells present in the human large intestine. Furthermore, gut-on-a-chip was used as a-proof-of-concept targeting Human coronavirus NL63 (HCoV-NL63), as a model of SARS-CoV-2 infection for drug testing 105. Nafamostat, a protease inhibitor, was observed to reduce viral entry, likely via inhibiting the host TMPRSS2 receptor while remdesivir was toxic to the endothelium 105. Overall, microfluidic platforms open the doors for investigating secondary and systemic drug toxicity in the physiological microenvironment 106, 107. Repurposing FDA-approved drugs for antiviral treatments are the economical and expedient strategy to confront COVID-19 infection.

## Emerging direction to confront the SARS-CoV-2 variants of concern

SARS-CoV-2 is continuing to evolve across the globe, engendering variants with increased transmissibility and pathogenicity 1. Several types of viruses, especially enveloped viruses, spread via cell-to-cell interaction; thus, surface topography may guide the spread of virions 108. The development of pre-clinical models of human viral infection that employ human cell-based biomimetic microfluidic platform is effective to elucidate cell-to-cell virus spreading, specific host response to infection, and immune cell recruitment to predict new antiviral agents. The FDA is piloting the use of tissue chips as possible drug testing tools, through the Innovative Science and Technology Approaches for New Drugs (ISTAND) Pilot Program 108.

With the resurgence of the SARS-CoV-2 variants, there is a persistent need to leverage the microfluidic technology to design new therapeutic drugs 43. Researchers have been investigating the mechanisms underlying a wide array of COVID-19-associated symptoms 43. SARS-CoV-2 is reported to mimic over 150 of its host proteins; most promote vascular inflammation and blood coagulation 65. Also, viral tropism for infecting numerous organ systems warrants the development of a multi-organ-on-a-chip (MoC) to rapidly address immunogenicity and pathogenicity 12. Current studies of viral infection using OoC are based on the use of individual microfluidic platform; thus, rendering it challenging to determine secondary and systemic toxicity 19, 46. While MoCs offer an appealing alternative to mimic both *in vivo* and *in vitro* studies, big data analysis to predict the therapeutic targets remains an unmet clinical need for personalized medicine 46.

Future direction will require efficient evaluation of a host of drugs and compounds via the use of high-throughput cell-based assays with SARS-CoV-2-on-a-chip. Additional effort is needed to understand how the human body responds to SARS-CoV-2 and drug therapies. Drug delivery, efficacy, and toxicity are the three pivotal aspects in the context of immune response in COVID-19 patients. For this reason, drug encapsulation in protein-coated nano-liposomes allows for reduction in drug dosing, toxicity, and high selectivity for specific cellular or viral targets 88, leading the way to alternative routes of administration, such as inhalation.

The integration of nano-liposome-based drug delivery and microfluidic platforms paves the way for bridging the current knowledge gap in tissue-specific response to SARS-CoV-2 and external stimuli. Overall, engineering viral genomics in the tissue-specific microfluidic platforms usher in an effective strategy to address sample size, power analysis, and quality control. This is essential for scaling up the analyses of SARS-CoV-2 variants and therapeutic targets in the long COVID-19 era.
